# Simulated and Experimental Time-Resolved Photoelectron Spectra of the Intersystem Crossing Dynamics in 2-Thiouracil

**DOI:** 10.3390/molecules23112836

**Published:** 2018-11-01

**Authors:** Sebastian Mai, Abed Mohamadzade, Philipp Marquetand, Leticia González, Susanne Ullrich

**Affiliations:** 1 Institute of Theoretical Chemistry, Faculty of Chemistry, University of Vienna, Währinger Straße 17, 1090 Vienna, Austria; sebastian.mai@univie.ac.at (S.M.); philipp.marquetand@univie.ac.at (P.M.); 2 Department of Physics and Astronomy, University of Georgia, Athens, GA 30602, USA; Abed.Mohamadzade@uga.edu

**Keywords:** DNA, photochemistry, excited states, time-resolved photoelectron spectroscopy, simulation

## Abstract

We report time-dependent photoelectron spectra recorded with a single-photon ionization setup and extensive simulations of the same spectra for the excited-state dynamics of 2-thiouracil (2TU) in the gas phase. We find that single-photon ionization produces very similar results as two-photon ionization, showing that the probe process does not have a strong influence on the measured dynamics. The good agreement between the single-photon ionization experiments and the simulations shows that the norms of Dyson orbitals allow for qualitatively describing the ionization probabilities of 2TU. This reasonable performance of Dyson norms is attributed to the particular electronic structure of 2TU, where all important neutral and ionic states involve similar orbital transitions and thus the shape of the Dyson orbitals do not strongly depend on the initial neutral and final ionic state. We argue that similar situations should also occur in other biologically relevant thio-nucleobases, and that the time-resolved photoelectron spectra of these bases could therefore be adequately modeled with the techniques employed here.

## 1. Introduction

The photophysics of the five canonical nucleobases of DNA and RNA—adenine, cytosine, guanine, thymine, and uracil—has attracted considerable attention in the last decades [1,2,3,4,5,6]. The reason is that these nucleobases are the principal chromophores of DNA/RNA in the UV range of sunlight. Absorption of UV photons can promote these molecules into electronically excited states, making them very reactive such that they can in principle undergo photochemical reactions with the surroundings, thereby damaging the DNA. One of the most exceptional properties of the five canonical bases is, however, that they can relax back to the electronic ground state nonradiatively within a few picoseconds, avoiding any photoreactions and thus protecting the DNA.

Besides the five canonical nucleobases, in nature, one can also find a large number of similar heterocycles, which are called nucleobase analogues [7,8,9]. Due to their similar structure, in the ground state, they exhibit a chemistry comparable to the canonical nucleobases. A consequence is that some nucleobase analogues are even incorporated in DNA in place of the canonical ones [7], or are present in different types of RNA [10,11], where they might have played an evolutionary important role [11]. One of the most actively investigated class of nucleobase analogues are the thio-nucleobases (thiobases), where an oxygen atom of a canonical base is replaced by sulfur. These compounds have some pharmaceutic [12,13,14,15] and photo-chemotherapeutic [16,17,18] applications because they exhibit a very interesting photophysics involving ultrafast intersystem crossing (ISC) with near-unity quantum yields [8,9,19].

One example of biologically relevant thiobases is 2-thiouracil (2TU). Its photophysics was previously investigated in solution with μs/ns [20,21,22] and fs [23,24,25] transient absorption spectroscopy and with fs transient vibrational spectroscopy [26]. In the gas phase, previous experiments were performed with time-resolved photoelectron spectroscopy (TRPES) [27,28] with multi-photon ionization by some of us, and recently were confirmed by Townsend and coworkers [29]. This large set of experimental evidence has been complemented by several theoretical studies on the excitation spectrum [30] and on the excited-state potential energy surfaces and relaxation pathways [31,32,33,34]. Furthermore, its excited-state dynamics has been simulated [35,36] with surface hopping techniques and the multi-state complete active space second-order perturbation theory (MS-CASPT2) [35] and algebraic diagrammatic construction (ADC(2)) electronic structure methods [36].

In this work, we present the first TRPES measurements of 2TU which employ single-photon ionization to probe the excited-state populations of the molecule during its dynamics. Although this technique can suffer from increased noise sources in the signal (photoelectrons from stray photons, pulse coherences, probe-pump signals) compared to multi-photon ionization, it offers the advantage of more intuitive interpretation and easier theoretical modeling. These experimental results are complemented by the simulation of time-dependent photoionization spectra based on the nonadiabatic trajectories presented in Ref. [35]. In order to describe the photoionization process, we apply computationally efficient procedures previously reported by several groups [37,38,39,40,41]. We use the computed time-resolved spectrum and its state-wise decomposition to investigate how the experimentally observed time constants are related to the intrinsic dynamics of the electronic populations, as obtained in the trajectory simulations. We show that, in 2TU, already simplistic approaches for computing time-resolved photoelectron spectra work well, which is due to the favorable properties of the excited and ionic states of this molecule, i.e., all neutral states correlate with the first two ionic states.

## 2. Experimental Details

The TRPES experiments use a femtosecond laser system with UV conversion capabilities, magnetic bottle photoelectron spectrometer, and a gaseous molecular beam source. More details about the apparatus have been described previously [42,43,44,45,46,47], while here only a short description is given. A Coherent Inc. (Santa Clara, CA, USA) amplified Ti:Sa laser system (Mira Optima 900F, Legend Elite) was used to pump two optical parametric amplifiers (OPerA and TOPAS-C) to generate pump and probe pulses. The OPerA was set to 293 nm (6 μJ energy per pulse) and served as a pump pulse to excite 2TU into the first absorption band. A second set of experiments was carried out using excitation at 260 nm, where the pump energy was 2 μJ per pulse. A deep-UV probe pulse facilitates one-photon ionization and is generated from the UV output of the TOPAS-C mixed with the amplifier fundamental in an external, custom-built sum-frequency generation setup. Specifically, the TOPAS-C is tuned to 257 nm (30 μJ) and its output is overlapped in space and time inside a β-barium borate (BBO) crystal with pulses of about 500 μJ from the fundamental (800 nm). The probe pulse is passed through an optical delay line for manual control of the relative timing and is mildly focused by a 2 m lens to enhance conversion efficiency. This configuration yields 2.0 μJ pulses of 194 nm, which are attenuated to 0.6 μJ to avoid contributions from multi-photon ionization in the TRPES scan.

The pump and time-delayed probe pulses are focused into the ionization region of the spectrometer by individual 50 cm lenses and spatially overlapped. Care is taken to minimize any stray light from the deep-UV probe which accounts for most of the unwanted background photoelectron signals. Measures include the use of a light baffle on the exit window of the chamber and avoiding scattering centers (e.g., burn spots, dust or impurities) on the entrance window. The 2TU powder is heated up to 205 ${}^{\circ }$C in a quartz sample holder inside the nozzle and a continuous molecular beam based on a He backing gas carries the evaporated sample into the ionization region of the spectrometer.

The time of zero pump-probe delay and the instrument response function were defined by TRPES measurements of a calibration gas consisting of a 50:50 mixture of 1,3-butadiene and He. At the pump and probe wavelengths employed in this study, Gaussian cross-correlation functions with a full width at half maximum (FWHM) of about 275 fs were measured, suggesting FWHMs of about 195 fs for each of the pump and probe pulses.

The magnetic bottle operates on the principle of a time-of-flight measurement. The same 1,3-butadiene/He gas mixture serves for energy calibrations, where known peaks in the photoelectron spectrum of butadiene are used to determine the instrument-specific calibration curve that relates measured photoelectron flight times to their kinetic energies [48]. Different positive and negative voltages are applied to the acceleration region to shift the butadiene peaks across the relevant time-of-flight range in order to increase the accuracy of the calibration.

Two-dimensional TRPES scans of 2TU are recorded over pump-probe delays from approximately −1 to 4 ps with a step size of 25 fs, which covers the range most relevant to the theoretical simulations where spectral changes in the photoelectron spectrum occur. The 2D-TRPES data is deconvoluted into its decay dynamics and associated spectra (DAS) using a global analysis program [42,43]. The fitting procedure was limited to optimizing and extracting the short time dynamics, while the long-lived time constant was fixed to previously reported values (i.e., an exponential decay constant of 203 ps for 293 nm excitation [27,28], or 86 ps for 260 nm excitation [28]).

## 3. Computational Details

For the simulations of the TRPE spectrum of 2TU, 44 trajectories were taken from Ref. [35], computed with the surface hopping including arbitrary couplings (Sharc) method and the multi-state complete active space second-order perturbation theory (MS-CASPT2) electronic structure level of theory, as detailed below.

### 3.1. Excited-State Dynamics Simulations

The electronic structure calculations for the dynamics simulations were performed with MS-CASPT2 [49,50] with an active space of 12 electrons in nine orbitals (8 π and π* orbitals from 8 non-hydrogen atoms, plus the lone pair of sulfur). This choice of active space has been shown before [27,33] to give very similar results as a much larger CAS(16,12) computation, and hence is regarded as an adequate choice for the present simulations. The orbitals were obtained with CASSCF(12,9), with state-averaging including either four singlet or three triplet states. We used the Dunning cc-pVDZ basis set [51,52]. Scalar relativistic effects were described with the second-order Douglas–Kroll–Hess (DKH) Hamiltonian [53], while spin-orbit couplings (SOC) were computed with the RASSI [54] and AMFI [55] formalisms. In the CASPT2 step, the IPEA shift (ionization potential-electron affinity shift) [56] was set to zero [33,57]. Intruder states were avoided with an imaginary shift of 0.1 Hartree [58].

Initial conditions (nuclear geometries and velocities) were sampled from the Wigner distribution of the lowest vibrational state of the ground state [59,60], based on a normal mode analysis at the MP2/cc-pVDZ level of theory. At each of the sampled geometries, a single-point calculation at MS-CASPT2(12,9) level of theory delivered excitation energies and oscillator strengths. From this data, the initial electronic states were selected [61] in the excitation energy window between 3.9–4.2 eV, hence all starting in the $S_{2}$ ($\pi_{S}\pi$*) state.

The trajectory simulations were performed with the Sharc method [62,63], including three singlets and three triplets (treating triplet components separately) in the dynamics. The nuclei were propagated with the velocity-Verlet algorithm with a time step of 0.5 fs, while the electronic wave function was propagated using the local diabatization formalism [64,65]. We employed an energy-based decoherence correction scheme [66] (with parameter of 0.1 Hartree [66]). These simulations produced 44 trajectories, with simulation time up to 1000 fs, that were reported in Ref. [35].

### 3.2. TRPES Simulations

In order to simulate the TRPE spectrum, the energies of each trajectory were recomputed at each 10th time step (every 5 fs), where additionally five ionic doublet states were calculated with MS-CASPT2(11,9)/cc-pVDZ. Based on the CI (configuration interaction) vectors of the perturbatively modified CASSCF states [50], Dyson norms were computed for all singlet-doublet and triplet-doublet state pairs (30 pairs in total). Dyson norms were computed with the WFoverlap program [67], which calculates exact wave function overlaps and Dyson orbitals in non-orthogonal molecular orbital bases. The Dyson orbitals are defined as:

(1) $$|\phi_{IF}^{D}\rangle =\sqrt{N}{\langle \Psi_{F}^{\left(N-1\right)}|\Psi_{I}^{\left(N\right)}\rangle }_{\left(N-1\right)},$$

where *I* is the initial neutral state and *F* is the ionic final state; integration in the braket is carried out over the coordinates of N−1 electrons. The norm of these orbitals, which we abbreviate as $|D_{IF}|$, is always between 0 and 1, and is a measure for the compatibility of the neutral and ionic states regarding a single-electron removal—a high Dyson norm is an indicator for a large photoionization probability and vice versa. As the initial state *I* in the Sharc simulations is actually a linear combination of several singlets and triplets—which is a basic paradigm in Sharc—the computed Dyson norms between singlets, doublets, and triplets were appropriately combined to yield the Dyson norms from the initial state (the active state) to all of the doublet states.

As a result of these computations, for each of the trajectories *j* and for each time step $t_{i}$, the following data was obtained: (i) ionization energies $\Delta E_{IF}^{j}\left(t_{i}\right)$ between the currently occupied neutral state *I* and each of the different ionic states *F* (note that these values are shifted by the excitation energy $E_{\mathrm{exc}}\left(t_{0}\right)$ to yield total binding energies; see below); and (ii) Dyson norms between these states $|D_{IF}^{j}\left(t_{i}\right)|$. Using this data, we computed the photoelectron spectrum $S(t,E_{b})$, where *t* is the time delay between excitation and ionization and $E_{b}$ is the total binding energy [27,28], which we define as:

(2) $$E_{b}\left(t\right)=\underset{\Delta E_{IF}\left(t\right)}{\underset{︸}{E_{\mathrm{ion}}\left(t\right)-E_{\mathrm{neutral}}\left(t\right)}}+\underset{E_{\mathrm{exc}}\left(t_{0}\right)}{\underset{︸}{E_{\mathrm{bright}}\left(t_{0}\right)-E_{S_{0}}\left(t_{0}\right)}}.$$

Here, $E_{\mathrm{neutral}}\left(t\right)$ and $E_{\mathrm{ion}}\left(t\right)$ are the energies of the occupied neutral and target ionic states, respectively, and $E_{\mathrm{bright}}\left(t_{0}\right)-E_{S_{0}}\left(t_{0}\right)$ is the initial excitation energy. Functionally, this is equivalent to the following definition of the binding energy in the experimental spectra:

(3) $$E_{b}\left(t\right)=E_{\mathrm{pump}}+E_{\mathrm{probe}}-E_{\mathrm{kin}}\left(t\right),$$

where $E_{\mathrm{kin}}\left(t\right)$ is the kinetic energy of the detected photoelectrons.

In order to compute the time-dependent photoelectron spectrum, we employ an intuitive convolution approach, which has been reported in the literature several times before [37,38,39,40,41]. For each trajectory *j* and each time step $t_{i}$, the contribution to the photoelectron spectrum $S^{j}\left(t_{i},E_{b}\right)$, depending on the binding energy $E_{b}$, is computed as:

(4) $$S^{j}\left(t_{i},E_{b}\right)=\sum_{F}^{\mathrm{ionic}}\sigma_{IF}^{j}\left(t_{i},E_{b};E_{\mathrm{probe}}\right)\Omega \left(E_{b},\Delta E_{IF}^{j}\left(t_{i}\right);\varepsilon \right),$$

where the sum runs over all ionic final states *F*, and *I* is the initial state (the active state in the diagonal representation for trajectory *j* at time step $t_{i}$). The cross-sectional term $\sigma_{IF}^{j}\left(t_{i},E_{b};E_{\mathrm{probe}}\right)$ describes the intensity of the transition between *I* and *F*, depending additionally on the energy of the probe laser $E_{\mathrm{probe}}$. Due to the finite (and point-like) trajectory ensemble, we also require a line shape function $\Omega (E_{b},\Delta E_{IF}^{j}\left(t_{i}\right);\varepsilon )$, which is located at the energy of the I→F transition, $\Delta E_{IF}^{j}\left(t_{i}\right)$, and whose width is governed by the parameter ε [41]. The latter should be chosen smaller than the width of the spectral bands but broad enough to smooth any arbitrary fine structure solely arising from the finite ensemble of trajectories.

In order to evaluate these contributions, we make two basic assumptions. The first is concerned with the photoionization cross section, which for any trajectory and time step can be written as [41,68]:

(5) $$\sigma_{IF}\left(E_{b};E_{\mathrm{probe}}\right)=\frac{4\pi^{2}}{c}kE_{\mathrm{probe}}|\langle \phi_{IF}^{D}|\vec{\mu }\cdot \vec{u}|\Psi_{F}^{\mathrm{eject},k}{\rangle |}^{2},$$

where $k=\sqrt{2(E_{\mathrm{probe}}-E_{b})}$ is the momentum of the ejected electron, $\vec{\mu }\cdot \vec{u}$ is the scalar product of dipole operator and unit vector in polarization direction of the laser, and $\Psi_{F}^{\mathrm{eject},k}$ is the wave function of the ejected electron (which depends on *k*). This expression can be obtained within the dipole approximation and the strong orthogonality approximation [68]. The dipole approximation is justified here because the wave length of light of less than 10 eV (larger than 120 nm) is much longer than the spatial extent of the molecule (about 1 nm). Here, our first assumption is that the cross section can be simplified to:

(6) $$\sigma_{IF}\left(E_{b};E_{\mathrm{probe}}\right)\approx C{\left|\langle \phi_{IF}^{D}|\phi_{IF}^{D}\rangle \right|}^{2},$$

where *C* is some arbitrary constant and the squared term is the Dyson norm mentioned above. With this assumption, we ignore the influence of the wave function of the ejected electron. This approximation is regularly done in the simulation of TRPE spectra, as the computation according to Equation (5) is very expensive; however, it has been shown previously that using Dyson norms as measures of photoionization probability often works quite well [69], even in the case of small photoelectron kinetic energies in simulating TRPE spectra [41]. Besides using Dyson norms, for comparison purposes, below we will also show TRPE spectra simulated when setting all relevant Dyson norms between active state and ionic states to 1, such that any dependence of the photoionization cross section on time, geometry, or state characters is excluded. Then, the time-dependence of the simulated spectrum only relates to the evolution of the energy gaps between active neutral state and ionic states.

The second assumption that we make is related to the line shape function, and, therefore, implicitly, to the change of vibrational energy during the ionization step. Here, our line shape function is based on the ideas of Fuji et al. [70] and Arbelo-González et al. [41] and is given by a rectangle function:

(7) $$\Omega \left(E_{b};\Delta E_{IF}^{j}\left(t_{i}\right),E_{\mathrm{probe}}\right)=\left\{\begin{array}{c} 1\;\mathrm{for}\;\Delta E_{IF}^{j}\left(t_{i}\right)\leq E_{b}\leq E_{\mathrm{probe}},\\ 0\;\mathrm{otherwise}. \end{array}\right.$$

Note that this function does not depend on a width parameter ε but on the probe laser energy $E_{\mathrm{probe}}$.

Once the contributions to the photoelectron spectrum, $S^{j}\left(t_{i},E_{b}\right)$ have been computed, the total photoelectron spectrum can be obtained by summing the data over all trajectories *j* and performing a temporal Gaussian convolution to acknowledge that the experimental pulses have finite duration [37,38]. The total spectrum becomes:

(8) $$S\left(t,E_{b}\right)=\sum_{j}^{\mathrm{traj}}\sum_{i}^{\mathrm{steps}}\exp\left(-\frac{{\left(t-t_{i}\right)}^{2}}{2\tau_{\mathrm{laser}}^{2}}\right)S^{j}\left(t_{i},E_{b}\right),$$

where *j* runs over trajectories, *i* over time steps, and $\tau_{\mathrm{laser}}$ is the width of the instrument response function. For the spectra shown below, we employed a temporal broadening of 190 fs (FWHM), in line with the approximate width of the experimental probe pulse.

## 4. Results and Discussion

### 4.1. Experimental Results

**Figure 1:**
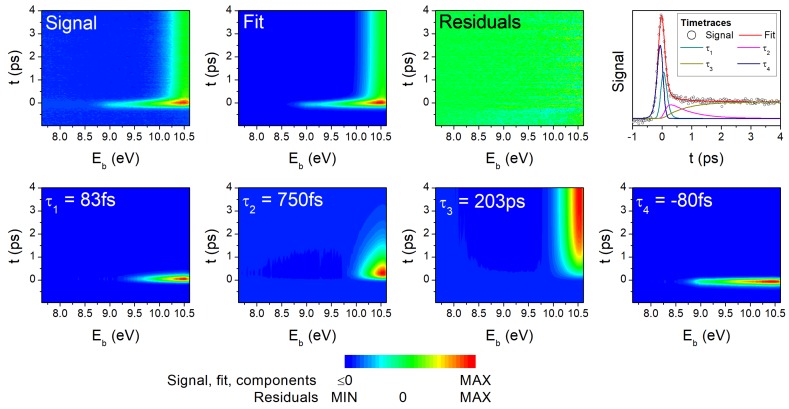
2D time-resolved photo-electron spectrum of 2-thiouracil recorded with 293 nm excitation and 194 nm one-photon ionization (**top row, first column**). Individual contributions to the 2D spectrum from global analysis techniques are plotted in the bottom row. Summation of these contributions yields the total fit (**top row, second column**) and when subtracted from the signal results in the residuals (**top row, third column**). All 2D spectra are plotted as color maps with the pump-probe delay, *t* (ps), along the *y*-axis and the electron binding energy, $E_{b}$ (eV), along the *x*-axis; signal intensities are represented according to the color bar on the bottom. The time traces (**top row, fourth column**) correspond to the signal, fit, and individual contributions integrated over all electron binding energies. Background subtraction errors in the probe-pump region amount to 3% of the maximum signal.

Figure 1 presents the TRPES data of 2TU recorded with 293 nm excitation and 194 nm one-photon ionization and its decomposition into individual DAS using global analysis techniques. A similar figure with the TRPES recorded with 260 nm excitation is shown in Figure S1 in the supporting information. A detailed description of the TRPES fitting procedure and interpretation of photoelectron spectra can be found in Refs. [27,28] and is only briefly described here.

The 2D TRPES shows an initial, intense and broad signal around time zero (t=0 ps region), which shifts towards higher electron binding energies within the first 1 ps and then stays constant without further spectral changes beyond the maximum pump-probe delay of 4 ps. According to Ref. [28], this long-lived signal decays with a time constant of 203 ps after excitation with 293 nm. Global analysis of the 2D TRPE spectrum requires three time constants to describe the sequential decay dynamics at positive pump-probe delays and an additional fourth time constant that accounts for probe-pump signals. The latter is due to strong absorption of 2TU at 194 nm, which results in unwanted signals from 194 nm excitation followed by 293 nm ionization that also contribute to the TRPE spectrum. Time constants for the fast dynamics of −80 fs ($\tau_{4}$), 83 fs ($\tau_{1}$) and 750 fs ($\tau_{2}$) were extracted from the fit, while the 203 ps ($\tau_{3}$) time constant [28] was kept fixed. The global analysis furthermore decomposes the TRPE spectrum into the different fitting components, which are plotted as color maps in the bottom row of Figure 1 (for the time-integrated DAS, see Section 4.3.3). The total fit (top row, second column) corresponds to the summation of all four contributions. Only random residuals (top row, third column) remain when the total fit is subtracted from the signal indicating that the decay model provides an adequate description.

The DAS serve for further analysis of the excited state relaxation pathway, which has been described in detail in Ref. [27] and is based on ionization correlations between the neutral excited and ionic states. According to ab initio calculations in Ref. [27], all neutral excited states—i.e., $S_{2}$ ($\pi_{S}\pi$*), $S_{1}$ ($n_{S}\pi$*), and the triplet states—preferentially ionize into either of the two lowest states of the cation (containing the $n_{S}$ hole or $\pi_{S}$ hole). Primarily, two factors contribute to the observed shifts of the DAS. First, there is a variation of the ionization potentials along the relaxation path, i.e., the ionization energies calculated at geometries of the different excited states minima. Second, the intramolecular vibrational energy increases during electronic relaxation to lower-lying states and the vibrational excitation is transferred to the cation upon photoionization. Accordingly [27], the photoelectron bands are expected to appear around 9.7 eV for ionization from the $S_{2}$ minimum, around 10.7 eV for the $S_{1}$ minimum, and around 10.1–10.9 eV for the two $T_{1}$ minima. Upon visual inspection of the DAS, the ultrafast contribution ($\tau_{1}$) can be assigned to ionization from the $S_{2}$ state and the intermediate contribution ($\tau_{2}$) to the $S_{1}$ state, which is also reinforced by the 1 eV shift of the photoelectron spectrum towards higher electron binding energies. Given the almost identical ionization energies of the lowest singlet and triplet states, no further spectral shifts are expected and the long-lived contribution ($\tau_{3}$) is therefore attributed to ionization from the triplet manifold. The lack of similar ab initio calculations for ionization from higher-lying neutral electronic states, prevents assignment of the probe-pump contribution ($\tau_{4}$), which, in any case, is of no significance to the present study.

### 4.2. Comparison of One- and Two-Photon TRPES Data

**Figure 2:**
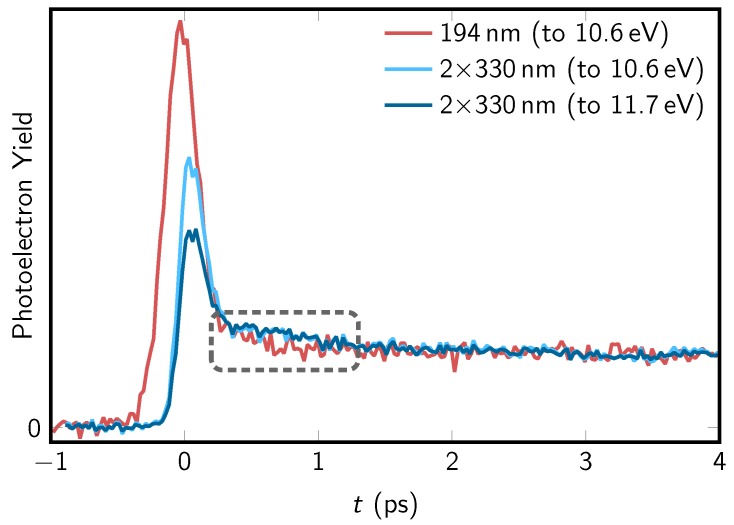
Time traces showing the total photoelectron yield as a function of pump-probe delay, *t* (ps). The TRPE spectra recorded at 293 nm + 194 nm (red) and 292 nm + 2 × 330 nm (light blue) were integrated over all electron binding energies $E_{b}\leq 10.6$ eV. The TRPE spectrum at 292 nm + 2 × 330 nm was also integrated for $E_{b}\leq 11.7$ eV (plotted in dark blue), i.e., over the entire range observable with the available (1 + 2’) total photon energy. All time traces are scaled so that their photoelectron yields match at long pump-probe delays in the 3–4 ps range. The grey box indicates the region where one- and two-photon experiments differ significantly.

In order to demonstrate the differences between the one- and two-photon TRPES data, in Figure 2, we plot the total (integrated) photoelectron yields as functions of the pump-probe delay. The one-photon data is shown in red and the two-photon ionization in blue. Note that the time traces are scaled such that the yields at long delays coincide.

For the two-photon ionization, we show two different curves, which were obtained by integrating to two different electron binding energy cutoffs. This is because, in TRPE spectroscopy, the excited-state dynamics are observed by projection onto the cationic states and, hence, the probe process is dependent on changes of the ionization potentials along the relaxation path. It is therefore conceivable that the observed decay dynamics and extracted time constants are affected by the total photon energy. The dark blue curve in Figure 1 corresponds to the photoelectron yield integrated from 7.8 to 11.7 eV, whereas the integration range of the light blue curve was truncated to 10.6 eV, comparable to the total photon energy available in the one-photon ionization data. The two time traces from two-photon ionization show notably different amplitudes of the initial peak around zero pump-probe delay. The difference arises because much signal intensity associated with the intermediate and long-lived contributions is located in the high-binding-energy range between 10.6 eV and 11.7 eV; hence, the spectrum integrated to 11.7 eV has a relatively weaker peak intensity at zero delay. Apart from this initial peak, the two plotted time traces from two-photon ionization closely resemble each other, which indicates that the signal for energies up to 10.6 eV provides all relevant information on the decay dynamics.

The one-photon ionization TRPE spectrum was also integrated to an electron binding energy cutoff of 10.6 eV and is displayed in Figure 2 as the red curve. In comparison to the two-photon data (light blue curve), the most obvious difference is the significantly stronger signal intensity around zero pump-probe delay. This is due to strong absorption of 2TU at 194 nm and contributions to the photoelectron signal associated with probe-pump processes, i.e., excitation with 194 nm and ionization with 293 nm, which extend towards negative delays.

A more subtle, but arguably more interesting, difference between the two time traces is observed in the 500–1000 fs region where photoelectron signal intensities are slightly lower for one-photon ionization. This particular region corresponds to the decaying signal of the $S_{1}$ ($n_{S}\pi^{*}$) state and the rising signal of the triplet states. As was shown previously [27], photoionization from the $S_{1}$ and $T_{1}$ minima requires high photon energies of about 10.7–10.9 eV at the optimized geometries. Nevertheless, the comparison between the 10.6 eV and 11.7 eV two-photon yields in Figure 2 demonstrates that it is not the total photon energy that is responsible for the difference between the one- and two-photon data. It is more likely that this difference is due to different selection rules for one- and two-photon ionization. According to our previous results [27,71] from the $S_{1}$ ($n_{S}\pi^{*}$) minimum ionization is preferentially into the $D_{1}$ state ($n_{S}$ hole). Oppositely, ionization from $S_{1}$ to the $D_{0}$ ($\pi_{S}$ hole) is weaker, although it is not forbidden due to the non-planarity of the $S_{1}$ minimum geometry. Now, in the case of two-photon ionization, it is conceivable that these selection rules are weakened, so that both $S_{1}\to D_{0}$ and $S_{1}\to D_{1}$ become more intense. The alternative, analogous explanation is that the triplet states exhibit less intensity with two-photon ionization than with one-photon ionization.

### 4.3. Simulated TRPE Spectra

#### 4.3.1. Overall Spectrum

**Figure 3:**
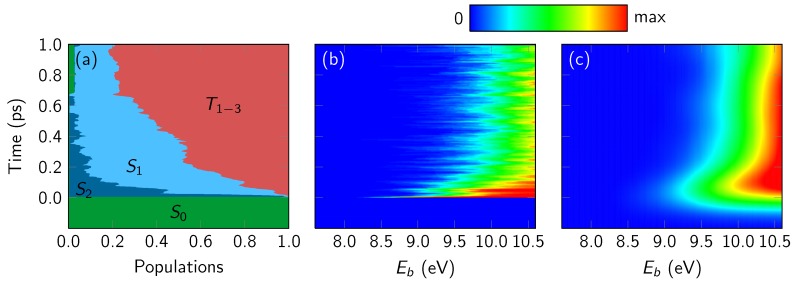
Electronic populations from the Sharc simulations (**a**) and simulated TRPE spectra without (**b**) and with (**c**) temporal broadening (full width at half maximum of 190 fs) added. $E_{b}$ is the binding energy, whose upper limit (10.6 eV) is equal to the sum of pump and probe energies (4.2 eV and 6.4 eV). The rectangular line shape function and intensities proportional to Dyson norms were used.

We begin the presentation of the simulated TRPE spectra of 2TU with Figure 3. It shows in panel (a) the electronic populations, as obtained from the 44 considered Sharc trajectories of Ref. [35]. In panels (b) and (c), we present the simulated TRPE spectra, using the rectangular line shape function and Dyson norms as intensity measure. Panel (b) shows the raw spectrum without temporal broadening, as it was directly produced from the trajectory data. Panel (c) shows the temporally broadened spectrum, using an FWHM of 190 fs. The spectra are plotted according to the binding energy $E_{b}$, which is computed as $E_{\mathrm{pump}}+\Delta E_{IF}^{j}\left(t_{i}\right)$. The maximum plotted binding energy corresponds to the combined energy of pump and probe lasers (4.2 and 6.4 eV, respectively) in the experiments.

Around time zero, the simulated TRPE spectrum in panel (b) shows a relatively strong signal which extends slightly below 9 eV, fitting well with the computed [27] ionization potential of 2TU with MS-CASPT2 (8.9 eV at the Franck–Condon point). This signal is due to ionization from the initially excited $S_{2}$ to the states $D_{0}$ to $D_{3}$, with the largest contributions from $D_{0}$ and $D_{1}$. Note that the simulated spectra include neither the cross-correlation peak—as this would necessitate the simulation of explicit laser-molecule interactions—nor the probe-pump signals. The latter would require trajectories launched after excitation with 6.4 eV, populating high-energy states which would not be described well with our MS-CASPT2 settings.

Within a few fs, the photoelectron band shifts above 9 eV, as the system is moving away from the Franck–Condon region. Subsequently, over the course of the first 100 fs, the signal loses much of its intensity. As already described in Ref. [27], the reason for this signal decay is primarily that the photoelectron band is pushed to higher binding energies due to the nuclear motion, such that most of the band becomes undetectable with the available laser energies. The shift of the band can be explained by two simultaneously occurring effects that affect the binding energy, which can be written as the sum of adiabatic ionization energy and vibrational energy gain [27] (see Equation (2) for a description of the individual terms):

(9) $$E_{b}\left(t\right)=\underset{\mathrm{adiabat}.\mathrm{IP}}{\underset{︸}{E_{\mathrm{ion}}\left(t\right)-E_{S_{0}}\left(t_{0}\right)}}\;+\underset{E_{\mathrm{vib}.}\mathrm{gain}}{\underset{︸}{E_{\mathrm{bright}}\left(t_{0}\right)-E_{\mathrm{neutral}}\left(t\right)}}\;.$$

By moving out of the Franck–Condon region and relaxing from $S_{2}$ to $S_{1}$, the system is converting excitation energy into nuclear vibrational energy that is not available for the ionization process. At the same time, the nuclear rearrangement in the $S_{2}$ and $S_{1}$ states leads to a destabilization of the ionic states.

The spectrum becomes stationary within 200 fs and stays approximately constant over the remaining simulation time, even though in this time the ISC process from $S_{1}$ to the triplet manifold (mostly $T_{1}$ and $T_{2}$) occurs. This can be understood from the fact that the $S_{1}$ and $T_{1}$ minima exhibit similar ionization potentials [27] and were predicted to have similar ionization spectra. This shows that, for 2TU, it is not trivial to distinguish the $S_{1}$ from the triplet states.

#### 4.3.2. State-Wise Decomposition

**Figure 4:**
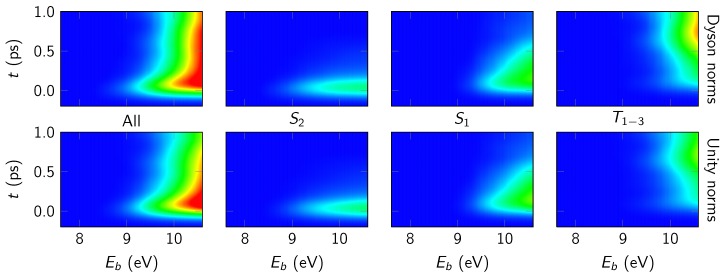
Simulated TRPE spectra of 2TU for the individual neutral states with temporal convolution (FWHM 190 fs). Each column of panels shows the spectral contribution of one initial (neutral) state ($S_{2}$, $S_{1}$, $T_{1-3}$), with the total sum given in the first column. The top row was computed with Dyson norms and rectangle line shape functions, the bottom row by setting all relevant Dyson norms to unity. As above, 4.2 eV pump energy and 6.4 eV probe energy were assumed in the computation of the binding energy. The color palette is the same as in Figure 3.

In Figure 4, we decompose the simulated spectrum according to the different active neutral states ($S_{2}$, $S_{1}$, $T_{1-3}$). The spectra in both rows were obtained with the rectangular line shape function and temporal convolution (FWHM of 190 fs).

We will first discuss the contributions of the individual states based on the top row of panels, which was obtained by considering Dyson norms as intensity measure. The results shown in the figure agree with the expectations from the populations in the dynamics simulation. The initially populated $S_{2}$ produces a signal only in the first 200 fs, whereas the $S_{1}$—which is populated quickly from $S_{2}$ and more slowly depopulated by ISC—produces an intermediate signal. The triplet states contribute strongly for longer simulation times. These results agree favorably with the decomposition of the experimental TRPES in Figure 1.

The figure also shows the spectra as obtained when setting all relevant Dyson norms equal to unity (labeled “Unity norms”) in the bottom row. Interestingly, the two sets of spectra (Dyson norms versus unity norms) agree very well with each other in this system. This observation is interesting because it means that the temporal and geometry dependence of the Dyson norms does not significantly affect the TRPE spectrum. A reason for this finding might be found in the correlation of the primarily involved neutral states ($S_{2}$, $S_{1}$, $T_{1}$) with the low-lying ionic states. In 2TU, $S_{2}$ is of ${}^{1}\pi_{S}\pi$* character, $S_{1}$ of ${}^{1}n_{S}\pi$* character, and the lowest two triplets are of ${}^{3}n_{S}\pi$* and ${}^{3}\pi_{S}\pi$* state. The two low-lying ionic states ($D_{0}$ and $D_{1}$) can be described as states with holes in either the $n_{S}$ or $\pi_{S}$ orbital. Hence, all involved neutral states correlate with either $D_{0}$ or $D_{1}$, and the sum of $X\to D_{0}$ and $X\to D_{1}$ photoionization probabilities is approximately constant independent of the neutral state *X*. This is true even if the molecular geometry becomes strongly nonplanar and the $n_{S}$ and $\pi_{S}$ orbitals mix. In this situation, it is apparently a good approximation to replace the Dyson norms by unit intensities.

#### 4.3.3. Time-Averaged Spectra

**Figure 5:**
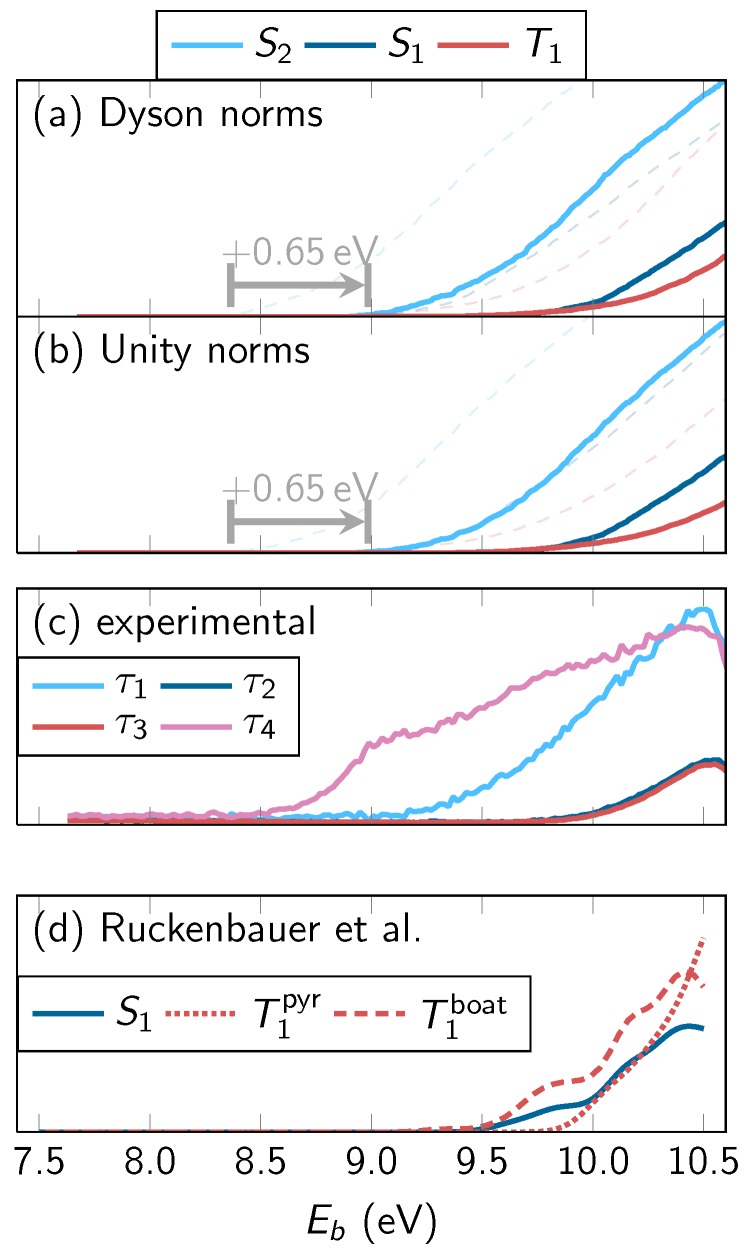
In (**a**,**b**), we show the simulated photoelectron spectra of each electronic state, computed by integrating over the whole simulation time, for Dyson norms and unity norms, respectively. For better comparison, each spectrum is normalized by the time-averaged population of each state and shifted by +0.65 eV to higher binding energies (the unshifted spectra are shown as thin, dashed lines). In (**c**), we show the experimental decay-associated spectra (DAS) for the four fitted time constants $\tau_{1}$ to $\tau_{4}$. The coloring of the four DAS was done in line with the assignment of $\tau_{1}$ to $S_{2}\to S_{1}$, $\tau_{2}$ to $S_{1}\to T$, and $\tau_{3}$ to $T\to S_{0}$. In panel (**d**), we also show the predicted ionization spectra of the vibrationally cold $S_{1}$ and $T_{1}$ minima from Ref. [71].

Figure 5 presents a comparison of the time-averaged simulated spectra of each neutral state with the DAS from the experiments and with the predicted spectra from Ref. [71]. For the simulated spectra, the lowest-energy spectrum is produced by the short-lived $S_{2}$ state, which is only populated directly after excitation, before vibrational relaxation sets in. The unshifted (thin, dashed lines) simulated spectra of $S_{2}$ have an onset of about 8.4 eV, slightly lower than the vertical ionization potential of 8.9 eV of 2TU at the Franck–Condon point [27] and slightly lower than the experimental onset of the spectrum at about 9 eV (panel (c)). This indicates that the method predicts energies for the vibrationally broadened ionization spectrum that are slightly too low. In order to compensate this shift, in the figure, the simulated spectra are shifted by +0.65 eV to higher binding energies.

The spectra of the $S_{1}$ and $T_{1-3}$ states are located at about 0.5–1.0 eV higher binding energies compared to the $S_{2}$ one. $S_{1}$ and $T_{1-3}$ produce partially overlapping spectra, where the triplet spectra are situated at only slightly higher energies than the $S_{1}$ spectra. This observation is consistent with the finding that the experimental DAS of $\tau_{2}$ and $\tau_{3}$ are nearly identical, except for a minor difference in intensity. We want to emphasize here that, even though these two DAS are nearly identical, from the experimental data, it is possible to distinguish two time constants due to the biexponential decay of the signal.

The spectra of $S_{1}$ and $T_{1-3}$ also qualitatively agree with the spectra simulated in Ref. [71]. However, unlike in the present results, Ref. [71] actually predicts lower energies for the triplet spectrum than for the $S_{1}$ spectrum. One of the main reasons for this disagreement might be that the spectra of Ref. [71] were computed assuming a cold vibrational distribution around the $S_{1}$ and $T_{1}$ minima. However, in the dynamics simulations, it appears that the $S_{1}$ is relatively hot and not yet equilibrated around the $S_{1}$ minimum, which leads to higher potential energies in the neutral states and thus lower binding energies. Another reason is that, in Ref. [71], only the $T_{1}$ was considered, whereas, in the dynamics simulations, some residual population of $T_{2}$ and $T_{3}$ contributes to the triplet spectrum.

#### 4.3.4. Energy-Integrated Yields

In order to compute time constants from the simulated TRPE spectra in Figure 3, we performed an integration over the energy axis. To this end, we used the data in Figure 3b because that approach is simpler and numerically more stable than fitting the broadened spectrum in Figure 3c with broadened fit functions. The integration range of the binding energy $E_{b}$ for the data was from 0 to 9.95 eV, which is the maximum binding energy in Figure 3b minus the shift of 0.65 eV determined above.

**Figure 6:**
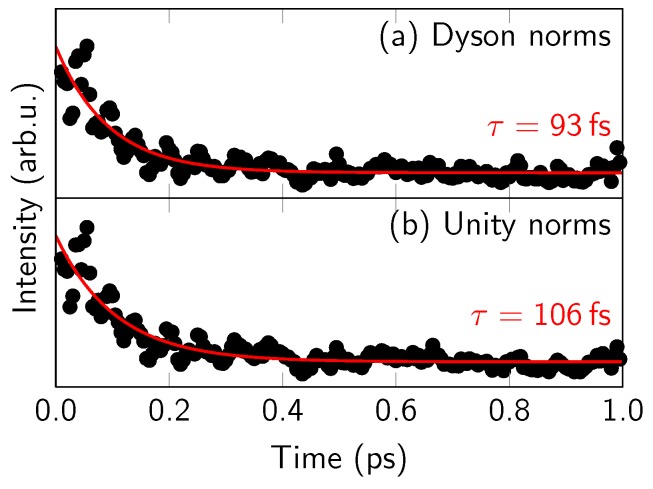
Integration of the simulated TRPES in Figure 3b for binding energies below 9.95 eV (i.e., 10.6 eV minus the estimated shift of 0.65 eV). Black dots show the integrated, temporally not broadened data, based on either Dyson norms or unity norms. The red curves are mono-exponential fits of the data, where the data in the first 20 fs (containing the large intensity spike at the Franck–Condon point) were excluded from the fit.

Figure 6 presents the obtained photoelectron yield over time, for both Dyson norms and unity norms. In both cases, the intensity drops by about 50% in the first 10 fs (not shown due to scale) as the system leaves the FC region. Then, the signal decays more slowly, with a time constant of about 100 fs. Consequently, the signals become approximately constant (except for noise) in the second half of the simulation time. Thus, from the total yields, it would be very difficult to extract the second time constant.

**Figure 7:**
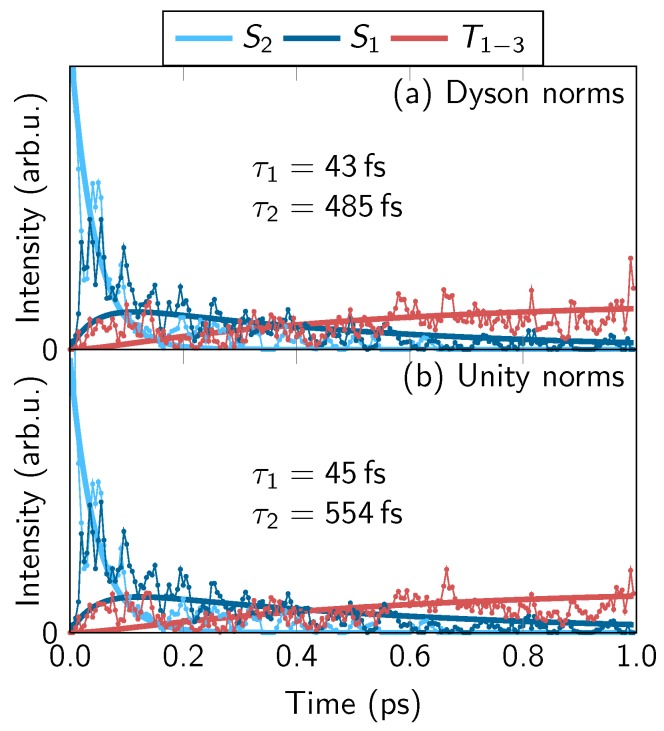
Integrated TRPES yields decomposed by state ($S_{2}$, $S_{1}$, $T_{1-3}$). The labels within the plots give the obtained time constant from fits with a three-component sequential kinetic model. Note that, in the fit, we assumed identical intensities for $S_{1}$ and *T*, based on the experimental intensities and the fact that the total yields are almost constant.

In order to still obtain two time constants that can be compared to experiment, we performed a decomposition of the yields into the contributions of the individual states. The results are shown in Figure 7, in a similar style as the fits of the experimental time traces in Figure 1 top right panel. The fit of the simulated TRPES yields is based on a sequential kinetic model $S_{2}\to S_{1}\to T_{1-3}$ and includes intensity prefactors for each of the states. However, we note that in the fit we kept the intensities of $S_{1}$ and $T_{1-3}$ identical.

From the fits, we obtain two time constants, $\tau_{S_{2}\to S_{1}}$ and $\tau_{S_{1}\to T}$. For both Dyson norms and unity norms, the first time constant is about 45 fs. This constant is consistent with the time constant of 60 fs obtained from the fit of the electronic population of the $S_{2}$ state [35], considering the fact that the energy differences between neutral and ionic states affect the time constants in Figure 7 but not the time constant from the electronic populations. Accordingly, this time constant can be attributed to internal conversion from $S_{2}$ to $S_{1}$.

The second time constant weakly depends on the use of Dyson or unity norms, and is around 500 fs. This time constant is slightly slower than could be expected from the electronic populations, which exhibit an overall ISC time constant of about 400 fs [35].

Interestingly, the fit of the state-wise decomposition of the TRPES yield gives significantly different results than the fit of the total yield, as seen in Figure 6. This can be explained by the similar intensities of the $S_{1}$ and $T_{1-3}$ states in our simulations, which lead to an approximately constant total yield for longer times.

### 4.4. Discussion

The relevant time constants for the final discussion are collected in Table 1. The most important of these data are the time constants $\tau_{1}$ and $\tau_{2}$, as these are the ones that can be compared between experiment and simulation. For $\tau_{1}$, which is assigned to the $S_{2}\to S_{1}$ internal conversion process, a general agreement is found for all experimental and theoretical values. The differences between the values can be fully explained by the error sources of $\tau_{1}$, like the experimental time resolution, temporal overlap with the probe-pump signal in the one-photon ionization experiments, and small errors in the employed MS-CASPT2 potential energy surfaces.

For the second time constant, $\tau_{2}$, the agreement between experiment and theory is slightly less satisfactory. The experimental value for excitation with 293 nm is 750–775 fs, as determined with two independent experiments (single- and two-photon ionization). The simulated value is shorter, with 400 fs based on the electronic populations and about 500 fs based on the fit of the decomposed TRPES in Figure 7. For this difference between experiment and theory, there can be several possible reasons. On one side, obtaining the experimental time constant from single-photon ionization is nontrivial due to the strong probe-pump signal and the noise due to stray electron background. While the probe-pump signal makes it difficult to cleanly fit the $\tau_{1}$ time constant, the noise in the data—especially in the range 200–1000 fs—partially covers the $\tau_{2}$ constant. In fact, it is possible to obtain a reasonable fit of the single-photon data presented in Figure 2 (red curve) when fixing $\tau_{1}$ and $\tau_{2}$ to the theoretical values. On the other side, the simulated time constants can be affected by inaccuracies in the electronic structure, dynamics method, and description of ionization. This is true even though in our previous publications [33,35] we extensively validated the electronic structure method against more accurate methods (MS-CASPT2 with much larger active spaces and basis sets). While this validation made sure that we obtain the qualitatively correct dynamics of 2TU, even very small variations on the order of 0.1 eV or less in the potential energy at critical points can have a quantitative effect on the decay times. For example, changing the excitation energy by 0.13 eV (from 4.25 to 4.38 eV) changes $\tau_{2}$ from 775 fs to 544 fs in the two-photon experiments [28]. Likewise, a small variation of the energy difference between $S_{1}$ minimum and $S_{1}/T_{2}$ crossing point—which is only 0.05–0.1 eV [33]—could also affect the ISC time constant significantly.

Another point that is worth mentioning here is the theoretical treatment of the ionization probabilities. Indeed, in the present work, we only employ two simple procedures to compute this quantity, either assuming constant intensities for all energetically allowed channels from the active neutral state to the doublet ionic states, or using Dyson norms to describe the ionization probability. Nevertheless, we obtain reasonable agreement with experiment, in particular very good integrated photoelectron spectra (Figure 5). This shows that Dyson norms can be used effectively to compute photoelectron spectra in certain cases, as has been reported previously by several authors [37,39,40,41]. The reason for this good description by these techniques is that the neutral and ionic states in 2TU lead to few important ionization channels, and those show very similar Dyson orbitals.

**Figure 8:**
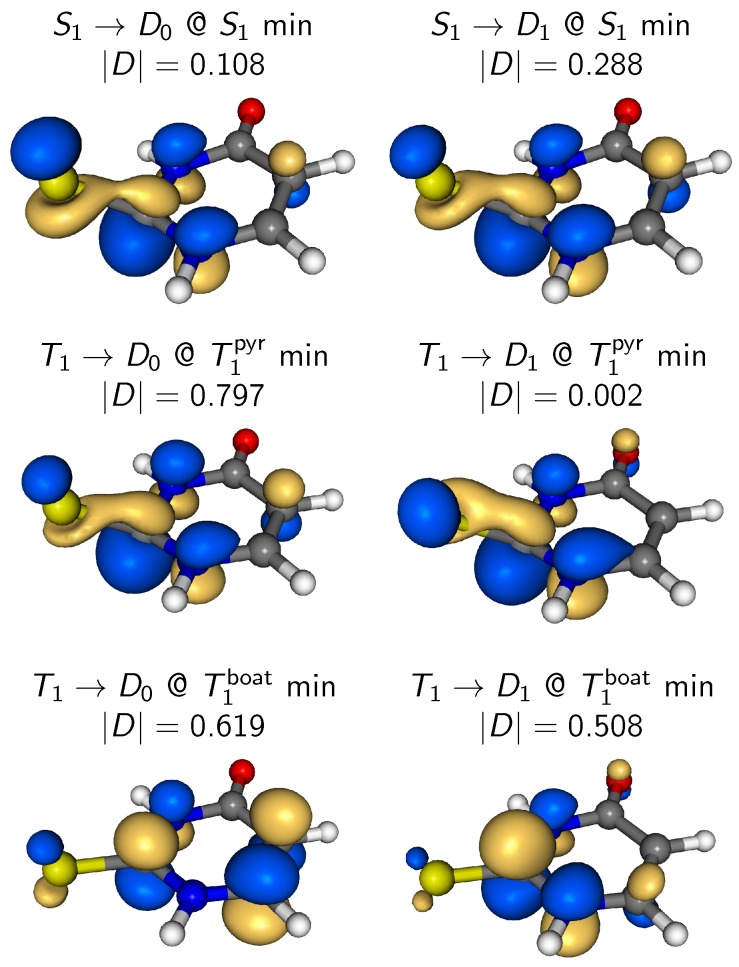
Dyson orbitals computed at the $S_{1}$ and $T_{1}$ minima of 2TU for ionization from $S_{1}$ or $T_{1}$ to $D_{0}$ or $D_{1}$. Note that, in order to show the renormalized Dyson orbitals, for each plot, we chose the isosurface value as $0.07\sqrt{\left|D\right|}$, where |D| is the relevant Dyson norm.

The Dyson orbitals for the most important neutral→ionic transitions for the ISC step—which mostly involves the $S_{1}$ and $T_{1}$ states around their minima—are shown in Figure 8. Because we want to emphasize the shape of the Dyson orbitals, in the figure, we plot the *renormalized* Dyson orbital by choosing an appropriate isosurface value for each orbital. Most notably, for almost all shown ionization channels the Dyson orbital has the same shape, being very similar to the $\pi_{2}^{*}$ orbital that is mostly localized on the carbon atom connected to the sulfur. The only exception is the $T_{1}\to D_{0}$ orbital for the boat-like $T_{1}$ minimum, being localized mostly on the C=C double bond. The reason for the very similar Dyson orbitals lies in the electronic structure, where the most important occupied orbitals are the sulfur $\pi_{S}$ and $n_{S}$ orbitals. The most important virtual orbital is the $\pi_{2}^{*}$, due to its stabilization at the pyramidalized geometries of the $S_{1}$ and $T_{1}$ minima. Since all low-lying neutral and ionic states ($S_{1}$, $T_{1}$, $T_{2}$, $D_{0}$, $D_{1}$) involve primarily transitions between these orbitals, there are very few possibilities how the Dyson orbitals can look like. The consequence of these very similar Dyson orbital shapes is that the photoionization cross section in Equation (5) can indeed be simplified to Equation (6) because the remaining factor *C*—which is $\langle {\bar{\phi }}_{IF}^{D}|\vec{\mu }\cdot \vec{u}|\Psi_{F}^{\mathrm{eject}}\rangle$, where the bar over ${\bar{\phi }}_{IF}^{D}$ denotes a normalized Dyson orbital (e.g., like the ones shown in Figure 8)—is approximately the same for all ionization channels.

Based on this discussion, we can now make predictions for the TRPES of other thiobases. In principle, the motif of two relevant occupied orbitals ($\pi_{S}$ and $n_{S}$) and one or two virtual orbitals can be found in all mono-thiobases, like 2-thiocytosine [19], 6-thioguanine [72], 4-thiothymine/4-thiouracil [73], 2-thiothymine [34], or 2TU. Hence, also for these thiobases, similar correspondences between neutral and ionic states are expected. This will be especially true for those thiobases that pyramidalize in the $S_{1}$ state—6-thioguanine [72], 2-thiothymine [34], 2TU, and 6-aza-2-thiothymine [74]—because the pyramidalization will stabilize only one of the virtual orbitals and thus simplify the ionization correspondences. Consequently, we suggest that the ionization yields of these thiobases along their relaxation pathways could also be simulated reasonably well and cost efficient with surface hopping or potential energy scans in combination with Dyson norm computations. This knowledge might be very useful for further studies of this biologically and medicinally important class of compounds. However, because the discussed electronic structure motif is directly connected to the thiocarbonyl group, we expect that this motif cannot be straightforwardly assumed for other heterocycles like the canonical nucleobases without further investigations.

## 5. Conclusions

To the best of our knowledge, we report the first time-resolved photoelectron (TRPE) spectra of gas phase 2-thiouracil using a single-photon excitation (293 nm) plus single-photon ionization (194 nm) scheme. Concomitantly, we also report computational simulations of these TRPE spectra, based on previously published [35] surface hopping trajectories at the MS-CASPT2 level of theory. Here, the use of single-photon ionization facilitates a better comparability of experiment and theory, as it is notoriously difficult to describe multi-photon ionization computationally.

The obtained experimental time constants are $\tau_{1}$ = 83 fs and $\tau_{2}$ = 750 fs (there is a third one of $\tau_{3}$ = 203 ps [28] that is beyond the scanning range), plus a negative time constant ($\tau_{4}$ = −80 fs) that is necessary to fit the probe-pump signal occurring through strong absorption of the probe laser by the ground state. The constants $\tau_{1}$ and $\tau_{2}$ are in good agreement with previously published TRPES experiments recorded with two-photon ionization [27,28], although with single-photon ionization the signal of $\tau_{2}$ is notably weaker. The computed time constants are $\tau_{1}$ = 45 fs and $\tau_{2}$ = 500 fs, based on a decomposition of the simulated photoelectron yield. Although the experimental and computed data agree qualitatively with each other, the slightly faster computed time constants are likely due to small inaccuracies in the employed potential energy surfaces.

The computed spectra show that for 2TU a simple description of ionization using Dyson norms is sufficient to obtain qualitatively correct results. Decompositions of the simulated spectra into contributions of $S_{2}$, $S_{1}$, and triplet states agree well with the fitted components from the experimental side. In addition, the time-averaged spectra agree very well with the DAS, except for a constant shift of about 0.65 eV. Our results show that the reason for the good performance of Dyson norm intensities is that the particular electronic structure of the neutral and ionic states. We have argued that these features of the neutral and ionic states are likely also present in other thio-nucleobases, which means that it should be possible to reproduce the TRPE spectra of these molecules with Dyson norms, instead of necessitating a more elaborate description of the ionization cross sections.

## Figures and Tables

**Table 1:** Time constants ${}^{a}$ for the excited-state dynamics of 2TU obtained with time-resolved photoelectron spectroscopy (TRPES) and nonadiabatic SHARC (surface hopping including arbitrary couplings) dynamics simulations.

Method	Pump (nm)	Probe (nm)	$\tau_{1}$ (fs)	$\tau_{2}$ (fs)	$\tau_{3}$ (ps)	$\tau_{4}$ (fs)	Remark
—experimental—							
Two-photon TRPES	293	2 × 330	<50	775	203		[27,28]
Two-photon TRPES	260	2 × 330	67	285	85.6		[28]
One-photon TRPES	293	194	83	750	203	−80	[present work]
One-photon TRPES	260	194	<50	246	85.6	−80	[present work]
—simulated—							
SHARC populations	295–317	none	∼60	∼400			[35]
Simulated TRPES	295–317	194	∼45	∼500			[present work]

${}^{a}$ Error estimates for the experimental time constants: 20% errors each for $\tau_{1}$, $\tau_{2}$, and $\tau_{3}$ of the two-photon experiments; 50% error for $\tau_{1}$ and 20% error for $\tau_{2}$ the one-photon experiments ($\tau_{3}$ was fixed to the two-photon values).

## Supplementary Materials

The following are available online at http://www.mdpi.com/1420-3049/23/11/2836/s1, Figure S1: TRPES recorded with 260 nm excitation.
