# Supplementary material for: Simulated and Experimental Time-Resolved Photoelectron Spectra of the Intersystem Crossing Dynamics in 2-Thiouracil
Source: Molecules. 2018 Nov 1;23(11):2836. doi: 10.3390/molecules23112836 (PMC6278540; doi:10.3390/molecules23112836)
Supplement: Supplementary file 1 [file molecules-23-02836-s001.pdf]

Supplementary Materials: Simulated and Experimental Time-Resolved Photoelectron Spectra of the Intersystem Crossing Dynamics in 2-Thiouracil

Sebastian Mai [1], Philipp Marquetand [1], Abed Mohamadzade [2], Leticia González [1],* Susanne Ullrich [2]*

TRPES using excitation at 260 nm

Figure S1 presents the TRPES recorded with excitation at 260 nm, in contrast to the TRPES shown in the main manuscript that employed 293 nm excitation. The main differences between the two spectra are that with 260 nm excitation (Figure S1), the second and third time constants are much smaller than with 290 nm excitation ($\tau_2 = 246$ fs instead of 750 fs; $\tau_3 = 85.6$ ps instead of 203 ps). Furthermore, in Figure S1 the shown residuals are slightly larger, which is due to stray light and background subtraction errors. However, the excited-state dynamics encoded by the TRPES shown in Figure S1 is qualitatively identical to the one discussed in the main text.

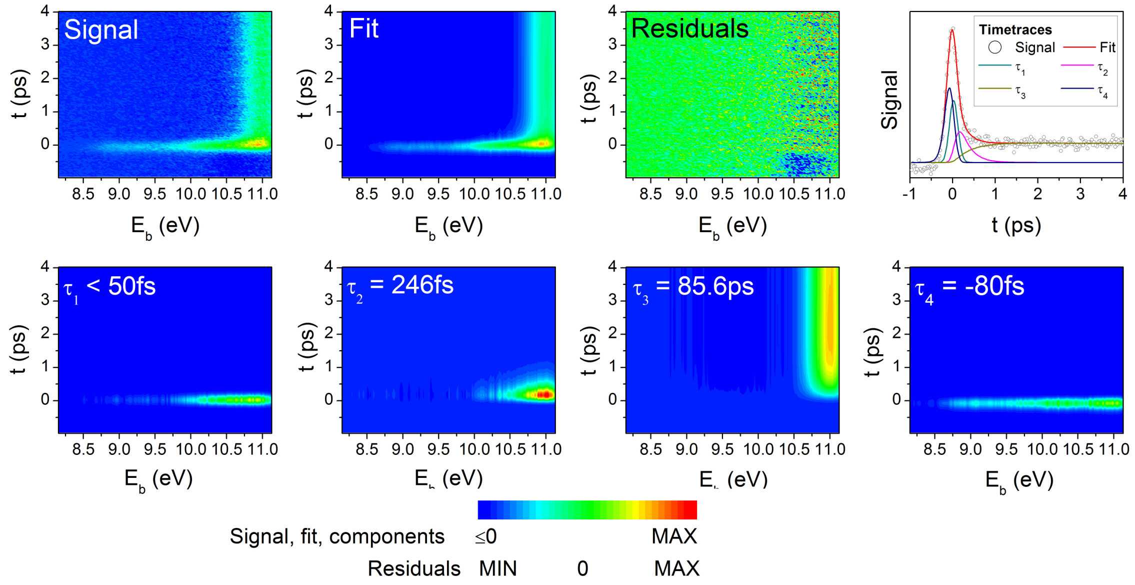

Figure S1. 2D TRPE spectrum of 2TU recorded with 260 nm excitation and 194 nm one-photon ionization (top row, first column). Individual contributions to the 2D spectrum from global analysis techniques are plotted in the bottom row. Summation of these contributions yields the total fit (top row, second column) and when subtracted from the signal results in the residuals (top row, third column). All 2D spectra are plotted as color maps with the pump-probe delay, t (ps), along the y-axis and the electron binding energy, E_b (eV), along the x-axis; signal intensities are represented according to the color bar on the bottom. The time traces (top row, fourth column) correspond to the signal, fit, and individual contributions integrated over all electron binding energies. Background subtraction errors in the probe-pump region amount to 5.4% of the maximum signal.
